# Motor skill depends on knowledge of facts

**DOI:** 10.3389/fnhum.2013.00503

**Published:** 2013-08-29

**Authors:** Jason Stanley, John W. Krakauer

**Affiliations:** ^1^Department of Philosophy, Yale UniversityNew Haven, CT, USA; ^2^Department of Neurology and Neuroscience, Johns Hopkins UniversityBaltimore, MD, USA

**Keywords:** motor skill, philosophy, HM, skill, knowledge

## Abstract

Those in 20th century philosophy, psychology, and neuroscience who have discussed the nature of skilled action have, for the most part, accepted the view that being skilled at an activity is independent of knowing facts about that activity, i.e., that skill is independent of knowledge of facts. In this paper we question this view of motor skill. We begin by situating the notion of skill in historical and philosophical context. We use the discussion to explain and motivate the view that motor skill depends upon knowledge of facts. This conclusion seemingly contradicts well-known results in cognitive science. It is natural, on the face of it, to take the case of H.M., the seminal case in cognitive neuroscience that led to the discovery of different memory systems, as providing powerful evidence for the independence of knowledge and skill acquisition. After all, H.M. seems to show that motor learning is retained even when previous knowledge about the activity has been lost. Improvements in skill generally require increased precision of selected actions, which we call *motor acuity*. Motor acuity may indeed not require propositional knowledge and has direct parallels with perceptual acuity. We argue, however, that reflection on the specifics of H.M.'s case, as well as other research on the nature of skill, indicates that learning to become skilled at a motor task, for example tennis, depends also on knowledge-based selection of the right actions. Thus skilled activity requires both acuity and knowledge, with both increasing with practice. The moral of our discussion ranges beyond debates about motor skill; we argue that it undermines any attempt to draw a distinction between practical and theoretical activities. While we will reject the independence of skill and knowledge, our discussion leaves open several different possible relations between knowledge and skill. Deciding between them is a task to be resolved by future research.

## Introduction

In Plato's *Gorgias*, Socrates draws a distinction between a *habitude* and an *art* (or craft, or skill). One mark of that distinction is that someone has mastery of an art in virtue of explicit knowledge of its first principles; otherwise it is merely a habit or tendency. As Plato writes (Plato, 1998):

I say it is not an art, but a habitude, since it has no account to give of the real nature of the things it applies, and so cannot tell the cause of any of them (465a).

On the view Socrates espouses in the *Gorgias*, someone with merely a reliable tendency, even an extremely reliable tendency, to obtain the desired results in a certain activity is not a true practitioner of that art. To be an expert, one needs to obtain the desired results in virtue of knowledge of the first principles of that activity. In fact, Socrates's position is even stronger than this: he seems to require a true practitioner of an art to be able to explain (“give an account”) of these principles.

Socrates's view in the *Gorgias* that expertise is constituted by explicit knowledge of first principles has not been treated kindly in the 20th century. In modern discussions of skill, it is the standard stalking horse. Even the less drastic view that skilled behavior is behavior guided by knowledge of an activity that starts out as explicit but eventually becomes implicit, is largely in disrepute. Both in cognitive neuroscience and much of philosophy, skills are thought not to involve knowledge of rules, but to belong in another category entirely.

The terms used to distinguish between practical and theoretical capacities, as will become clear below, are themselves disputed. Following standard nomenclature, we will (provisionally, and solely for the purposes of this paper) call a process “implicit” if learning proceeds without intention or awareness even if initiating, continuing, and practicing an action are intentional. Three thousand years of philosophy have not yielded a successful definition of knowledge. But knowledge is, minimally, a state with propositional content, one that is suitable for use in guiding action. As we will argue, whether or not the propositional content of a state can be verbalized (a test often used to distinguish between explicit and implicit knowledge) is irrelevant to its status as knowledge.

Gilbert Ryle's target in Chapter 2 of his immensely influential 1949 book *The Concept of Mind* is the view that “the primary exercise of minds consists in finding the answers to questions and that their other occupations are merely applications of considered truths” (Ryle, 1949). Ryle argued that skilled action was not *merely* the application of knowledge, whether explicit or implicit, but rather the manifestation of a non-propositional state that he labeled *knowledge how*. Ryle did not go as far as to deny that most skills required some knowledge in the classical sense; his position on such matters is unclear. However, subsequent philosophers have.

The philosopher Maurice Merleau-Ponty appears to deny the view that skilled behavior manifested cognitive states (Merleau-Ponty, 1962). Subsequently, Pierre Bourdieu uses Merleau-Ponty's work to argue that social practices, which are in effect networks of social skills, are habits in Plato's sense, and therefore independent of cognitive states. In recent years, Hubert Dreyfus has been the most influential and explicit of the anti-cognitivists. Summing up the anti-cognitivist position, Dreyfus writes (Dreyfus, 2005):

While infants acquire skills by imitation and trial and error, in our formal instruction we start with rules. The rules, however, seem to give way to more flexible responses as we become skilled. We should therefore be suspicious of the cognitivist assumption that, as we become experts, our rules become unconscious. Indeed, our experience suggests that rules are like training wheels. We may need such aids when learning to ride a bicycle, but we must eventually set them aside if we are to become skilled cyclists. To assume that the rules we once consciously followed become unconscious is like assuming that, when we finally learn to ride a bike, the training wheels that were required for us to be able to ride in the first place must have become invisible. The actual phenomenon suggests that to become experts we must switch from detached rule-following to a more involved and situation-specific way of coping.

Indeed, if learners feel that they can act only if they have reasons to guide them, this attitude will stunt their skill acquisition.

It is clear even from the very vocabulary of anti-cognitivists from Merleau-Ponty and Bourdieu (the “habitus”) through to Dreyfus that the pre-dominant 20th century tendency in philosophical and social scientific studies of skill is to reject Socrates's distinction between habitude and art. As Carlotta Pavese reminds us (Pavese, Unpublished), Ryle, ever knowledgeable about the tradition on the matter, went out of us way to distinguish between habits and skills (Ryle, 1949). It is not however completely clear if Ryle's positive view of the mental allows for the distinction the need for which he himself emphasizes. Knowledge of facts about the activity *hinders* expertise. It is hard to avoid the suspicion that the standard contemporary view it is only *mere* habitude in Socrates's sense that is genuine expertise. As Dreyfus and Dreyfus write (Dreyfus and Dreyfus, 1984):

The expert is simply not following any rules! He is doing just what Socrates … feared he might be doing – discriminating thousands of special cases.

It is quite natural to read much of the literature on the cognitive psychology and neuroscience of skill as mirroring much of the philosophical literature's repudiation of the view that skilled motor behavior is the application of knowledge. For example, there is evidence that active reflection on the principles of an activity impedes performance (Beilock et al., 2002). One could take this as further evidence for the non-cognitivist position. After all, if skilled behavior is guided by knowledge of facts about the activity, then it would seem that performance requires active reflection on such knowledge. But if “online attentional monitoring of step by step performance” hinders expert performance (Ibid.), then perhaps experts are not following rules at all.

The cognitive neuroscience literature seems to provide strong support for the view that at least retention of knowledge of facts about an activity is not required in order to be skilled at that activity. One of the central cases in cognitive neuroscience is the famous case of HM. According to the standard theoretical description of HM's deficit, he lost the capacity to retain knowledge of facts that he learned after the operation that caused his amnesia. However, he was supposedly able to acquire new procedural knowledge, and retain it (and even improve upon it) over time (Milner, 1962). Cognitive neuroscience often identifies procedural knowledge with the functional category of motor skill. The core idea that cognitive neuroscientists take from HM is that motor skills can be retained, even when knowledge about the activity fails to be retained. As a result, the standard view in cognitive neuroscience is that being skilled at an activity does not require knowledge of facts about that activity (Cohen and Squire, 1980).

Procedural knowledge is standardly opposed to *declarative knowledge*. Note that use of the word “knowledge” after procedural already introduces a contradiction. What is non-factual knowledge of an action? It is also rare to find any kind of clear characterization of declarative knowledge. But it is something like *knowledge that can be verbally articulated*. Another standard assumption is that declarative knowledge is knowledge in the “folk” sense of knowledge of facts, as well as the corresponding sense of knowledge relevant to traditional philosophical debates. The predominant tendency in the literature on motor skill is to seek to minimize cognitive aspects of its acquisition and improvement. It is commonplace to argue that explicit cognition is at best modulating or at worst contaminating motor learning. As a result, in some textbooks of cognitive neuroscience, motor skill is not included in the table of contents.

The consequences of this are significant. Sports are widely considered a motor activity and are often contrasted with intellectual or theoretical activities. This divide is apparent in the notion of the “dumb jock” and contentious debates about the role of sport on college campuses. Our main contention in this article is that this dichotomy has in part been driven by a misunderstanding of the scientific findings, exemplified by the case of HM. We will argue that this results from two overly hasty and ultimately incorrect identifications between *categories in neuroscience* and *mental kinds*. One is the identification of procedural knowledge, itself a misnomer, with the colloquial understanding of motor skill. The second is the identification of declarative knowledge with knowledge in the traditional sense.

Obviously, we do not hold the view that expertise at an activity requires explicit knowledge of first principles. Nevertheless, we reject the view that skill and knowledge are independent. In our view, skilled action is action guided by ongoing accrual and improving application of knowledge of facts about an activity, though skill is not exhausted by such knowledge. We will make the case that the use of the term “procedural knowledge” has misled because the term has been applied to those aspects of skilled performance that are arguably not based on knowledge. The intuitive appeal of the dichotomy between the practical and the theoretical is precisely because those aspects of motor tasks that have been called procedural may indeed not require knowledge of facts. But it does not follow from this that skills do not require knowledge of facts. In our view, an attribute of a component has been made to apply to the whole. Conversely, intellectual abilities likely ride on many implicit abilities that are not considered along with more obvious explicit processes (van Gaal et al., 2012).

We begin with a discussion of the pre-theoretic notion of skill, that is, the functional category that scientific accounts of skill should at the very least approximate. We argue that the actions most naturally thought of as manifestations of skill quite clearly are guided by knowledge of facts about that activity; we use as an example, *knowledge of what to do to initiate an action*. Of course, some manifestations of this knowledge are by agents who cannot verbally articulate its content. We also argue that this does not undermine its status as knowledge. Not only is the notion of “verbal articulation” that underlies declarative knowledge unclear, but there is no reason to make verbal articulation *in any sense* necessary for knowledge. We then turn to the case of HM (Milner, 1962). We argue that HM is not capable of acquiring motor skills. Just as it is a mistake to identify declarative knowledge with knowledge, it is a mistake to identify procedural knowledge with skill. Finally, we turn to other possible ways of grounding a distinction between skilled performance and theoretical activity on the findings of neuroscience, and argue that here too there is no hope of success. Cognitive neuroscience simply does not provide a basis for a distinction between the theoretical activities and practical ones, and deeper reflection on the matter in fact suggests that there is no such distinction to be drawn.

## Motor skill, perceptual discrimination and intention

What is the functional category that a theory of motor skill should explain? Let us begin with some cases. Consider the distinction between writing with your dominant hand, and writing with your non-dominant hand, and the distinction between throwing a baseball with your dominant hand and throwing a baseball with your non-dominant hand. Most of us are *skilled* in the former tasks, and *unskilled* with the latter ones. We are accustomed, by virtue of long training and experience, to write and throw baseballs with our dominant hands. We have no such training and experience with our non-dominant hands. To describe someone as “skilled” at an activity is to at least suggest that they have progressed far past baseline, or at least some contextually set point. But to describe someone has “having skill” at an activity does not bring with it the thorny issue of how far one must progress past baseline relative to such a contextually set standard. It is natural to assume that one necessary condition to have skill at φ-ing is *to have trained to be better than baseline at* φ*-ing*.

Having skill at returning a serve in tennis, pitching, swimming, and playing the piano are all ways in which one have a skill, whereby skill can be considered the practice-related improvement in a goal-directed action. Goal directed actions can be thought of as skilled both when the subject is better at accomplishing a task for rewards or points, and when their movements are deemed smooth, graceful and efficient. These are two different things—a gracefully hit shot can go out. A useful way about navigating the idea of motor skill is to compare and contrast it with perceptual abilities (sometimes misleadingly called “perceptual skills”), which can also improve with training, for example, discrimination for line orientation in peripheral vision (Watanabe et al., 2001). How is being better at discriminating line orientation different from returning a serve in tennis or pitching a curve ball in baseball? There are (at least) two differences.

First, though one can become *better than baseline* at discriminating the orientation of lines, the *basic* ability to perceive orientation does not require instruction nor does it need to be learned. In contrast, even the basic ability to return a serve in tennis or to pitch a curve ball in baseball must be instructed at least initially, or in some other way experientially learned, and further learning, either via instruction or observation, is required in order for training to proceed. That is to say, improvement is predicated on learning facts about the activity. This is a distinction between the two kinds of cases. Facial recognition, for example, falls on the perceptual learning side, and bicycle riding, for example, falls on throwing a curve ball side.

Here is a second distinction between the two kinds of cases, one that emerges quite clearly in one of Aristotle's discussion of the distinction between *craft* (skill) and *virtue* (Aristotle, 1999):

… in a craft, someone who makes errors voluntarily is more choiceworthy; but with prudence, as with the virtues, the reverse is true.

Aristotle's point is that is a mark of being skilled at an action is that one can make voluntary errors (and this distinguishes skill from virtue). As Aristotle writes in the Metaphysics [1046b]:

[1] it is clear that some of the potencies (dunameis) also will be irrational and some rational. Hence all arts, i.e., the productive sciences, are potencies; because they are principles of change in another thing, or in the artist himself qua other.

Every rational potency admits equally of contrary results, but irrational potencies admit of one result only. E.g., heat can only produce heat, but medical science can produce disease and health. The reason of this is that science is a rational account, and the same account explains both the thing and its privation, though not in the same way; and in one sense it applies to both, and in another sense rather to the actual fact. Therefore such sciences must treat of contraries—essentially of the one, and non-essentially of the other; for the rational account also applies essentially to the one, but to the other in a kind of accidental way, since it is by negation and removal that it throws light on the contrary. For the contrary is the primary privation, and this is the removal of that to which it is contrary. And since contrary attributes cannot be induced in the same subject, and science is a potency which depends upon the possession of a rational formula, and the soul contains a principle of motion, it follows that whereas “the salutary” can only produce health, and “the calefactory” only heat, and “the frigorific” only cold, [20] the scientific man can produce both contrary results. For the rational account includes both, though not in the same way; and it is in the soul, which contains a principle of motion, and will therefore, by means of the same principle, set both processes in motion, by linking them with the same rational account. Hence things which have a rational potency produce results contrary to those things whose potency is irrational; for the results of the former are included under one principle, the rational account.

Aristotle's point here is that our skilled actions are always under our rational control; a point that may have been lost to those of us now who appeal or seek to analyze the notion of skill in philosophy, cognitive psychology, and cognitive neuroscience, but it is front and center in Ryle's *The Concept of Mind*. As he writes (Ryle, 1949, p. 33), “The cleverness of the clown may be exhibited in his tripping and tumbling. He trips and tumbles just as clumsy people do, except that he trips and tumbles on purpose and after much rehearsal and at the golden moment and where the children can see him and so as not to hurt himself.”

It is natural to explain this otherwise puzzling feature of skill by appeal to the fact that manifestations of skill possession are (typically? invariably?) *intentional actions*. My playing the piano is a manifestation of my having skill at piano playing and the playing itself is an intentional action. It is an act about which, in Anscombe's famous characterization (Anscombe, 1963, p. 9), “a certain sense of the question ‘why’ is given application; the sense of course that in which the answer, if positive, gives a reason for acting.” Something is an intentional action, if it makes sense to ask the agent why he did it. The manifestations of what we would colloquially describe as skills are *paradigm examples* of intentional action, and are hence under our rational control. Take, for example, archery. An agent who has skill at archery manifests her skill by *deciding* to pick up a bow. That is, whether or not to manifest her skill is under her rational control. Carlotta Pavese's work (in particular her forthcoming paper, “Skills as Knowledge,” Unpublished) emphasizes this feature of skill, and discussions with her over the years have helped us to recognize its centrality and importance.

Paradigmatically, skills manifest in intentional actions. Conversely, paradigm cases of movements that are not intentional actions are also quite clearly not manifestations of skill; to give some of examples from Anscombe (1963, p.13), “the peristaltic movement of the gut,” and withdrawal of a hand “in a movement of involuntary recoil.” The direct manifestations of perceptual ability are also not intentional actions. Rather the manifestations of perceptual ability are *belief states*, the formation of which is generally regarded as not being under *direct* voluntary control (Williams, 1970). If a normally sighted agent perceives a table in front of him under normal lighting conditions, it's typically not possible for him to avoid forming the belief that there is a table in front of him. More generally, if one has strong evidence that *p*, it is difficult to avoid forming the belief that *p*.

As Bernard Williams (1970) notes, granting that belief is not under direct voluntary control, belief is nevertheless under *indirect* voluntary control. Perhaps I can voluntarily join communities that doubt the sources of evidence I have, and after long exposure, learn to reject such sources of evidence (such as the deliverances of climate science). Nevertheless, there remains a difference between capacities that directly and characteristically manifest themselves in belief formation, such as perception, and paradigm examples of skill. Whether or not to pick up a bow or a baseball bat is up to us in a way that forming a belief is not up to us, even though forming a belief may be *indirectly* up to us.

There are ways to argue that perceptual discrimination is not in the end as distinct along these two dimensions as they appear on the face of it to be. Having skill requires training past baseline. As a consequence, one can retain the view that having skill at discriminating line orientation requires learning. Even if the basic ability to discriminate line orientation is innate, having skill at it requires training past baseline, if not instruction. To address the second dis-analogy, one could also try to fold certain exercises of visual perception into the mold of intentional action. For example, I can selectively attend to portions of visual field. The fact that I can selectively attend could be used to argue that there is a kind of intentional action even in apparently passive faculties. Once I attend to the table, I cannot help but see it as brown, but it is up to me to decide to attend to the table in the first place.

However, this defense of perceptual discrimination as a skill is inadequate. First, selective attention is, like the methods Bernard Williams (1970) discusses, only a kind of *indirect* voluntary control. I can “decide” to form beliefs only about marbles by selectively attending only to marble-rich parts of my environment. But this is akin to “deciding” to form beliefs that the climate is not changing by attending Tea Party rallies. Secondly, there is ample evidence that perceptual ability can be acquired and improved in the *absence* of attention. For example, in a widely used motion-coherence detection task, subjects improved their discrimination abilities at a supra-awareness threshold version of the task having trained on a sub-awareness version of the task (Watanabe et al., 2001). If perceptual ability can be acquired and improved in the absence of attention, then its acquisition and improvement cannot be due to exercise the skill in (say) training, because one does not in these cases exercise even indirect voluntary control.

In the case of virtually any activity φ, having skill at φ-ing requires knowing what to do to initiate actions of φ-ing. Such knowledge is propositional knowledge (Stanley, 2011a, Chapter 2). Knowing what to do to initiate an action is clearly factual knowledge; it is the knowledge that activities x_1_ … x_n_ could initiate that action. It is a kind of factual knowledge required by skill possession. Furthermore, in the case of almost any complex skill, such as tennis, being skilled requires knowing to do to initiate a wide variety of actions. In other words, being skilled at an activity requires possession of a large amount of propositional knowledge about that activity.

Here is another way to see the point that being skilled at φ-ing requires propositional knowledge about the activity of φ-ing. In order to be skilled at φ-ing one must know how to φ. For example, being skilled at swimming requires know how to swim. Starting with Ryle (1949), there is an extensive 20th century tradition denying that knowing how to do something is factual (propositional) knowledge. That tradition has come under serious attack in recent years (Stanley and Williamson, 2001; Stanley, 2011b). Knowing how to swing one's racket seems to be the same kind of state as knowing when to swing one's racket, and knowing where to position one's racket. As Jennifer Nagel pointed out to us, knowing how to do something might stand in a task-subtask relation to other knowledge required for skill, such as knowing what to do to initiate the action (Personal Communication). One should have a uniform account of knowing-wh (knowing where, when, why, how, what, whether …). The most promising such account treats them all uniformly as factual knowledge. Knowing what to do to initiate an action and knowing how to do something are both kinds of factual knowledge, factual knowledge required by skill possession.

The expression “knowing what to do to initiate an action of φ-ing” contains what is known as an embedded question, the question “what to do to initiate an action of φ-ing.” This kind of construction is called “an infinitival question” (Bhatt, 2006, Chapter 4). Infinitival questions can only in English occur embedded inside factive verbs like “know” or “remember.” Infinitival questions have an ambiguity that is due to two different readings of the infinitive construction, “what to do.” As Stanley and Williamson (2001, p. 424) write:

So infinitives appear to have at least two different kinds of readings. On the first reading, they express deontic modality. In this case, a use of “to F” expresses something like “ought to F.” On the second reading, they express some kind of possibility. On this reading, a use of “to F” has a meaning that is similar to “can F”. These are the two readings that seem available for infinitives in embedded questions.

The sense of “knowing what to do to initiate action” in which it expresses a necessary condition on skill possession is the one in which the infinitive has an ability like modal reading. For x to have skill at archery, x must know what x could do to initiate an act of shooting a bow, e.g., x must know some of the things he could do to initiate an act of archery, such as (a requirement x may satisfy by knowing that he could start shooting arrows by fitting the arrow into the bow).

The claim that knowing what to do to initiate an action of φ-ing is a necessary condition for having skill at φ-ing does not entail that what to do to initiate an action is *always the same*. What to do to initiate an action of sailing differs from situation to situation, depending on weather and the landscape. Having skill in sailing is a state that requires having different knowledge states on different occasions, since knowing what to do to initiate an action at sailing will involve knowing one set of facts under stormy weather conditions, and another set of facts under calm weather conditions. As Carlotta Pavese has pointed out to us, x's knowing what to do to initiate φ-ing is having generic knowledge of the form: for situations s, x knows what to do to initiate an action of φ-ing in s (Pavese, Unpublished).

By “initiating an action” we do not mean starting a causal chain that terminates in an event, as choosing to eat some food is a voluntary action that starts a causal chain that terminates in a sequence of bodily events (these can be considered reflexes). Rather, we mean knowing what to do to begin an intentional action. (As Anscombe points out, the “peristaltic movement of the gut” is not action performed in the relevant sense by an agent, but only by her body; Anscombe, 1963).

Virtually anything colloquially describable as a “skill,” such as swimming, riding a bicycle, chess, basketball, and cooking, have agents who clearly know what to do to initiate the actions that manifest the skill. Part of having skill at throwing a curve ball is having the knowledge that throwing a curveball requires picking a baseball up (as well as knowing what to do with it when it is in your hand). Part of having the skill of chess is having knowledge about the starting moves of the game and its rules. In such cases, it is clear that part of having the skill is having the knowledge of what to do to initiate the action that manifests the skill. It should be emphasized that even starting conditions are complex, as in the sailing example above, and an expert will have a larger repertoire of starting actions and will know better how to apply them.

The claim that skill involves knowing what to do and how to do it explains Aristotle's comment about the distinctive nature of skill, which is that skills are under our voluntary control. Someone who has skill at φ-ing knows what to do to initiate an action of φ-ing; a skilled actor knows what she *could* do to start φ-ing. For example, someone who has skill at archery knows that she could start shooting arrows by fitting them into her bow. It is the modality associated with the infinitive that explains the distinctive nature of skill. If one can, in the relevant sense, start shooting arrows by fitting them into one's bow, and one knows that one can do this, then shooting arrows by fitting them into one's bow is under one's voluntary control. Thus, skill possession requires a kind of knowledge, possession of which entails voluntary control over one's actions. It is this feature of skill that explains its distinctive nature, and explains the feature of skill, that a mark of its exercise is the capacity to make voluntary errors, that is present in all classical discussions of skill from Aristotle to Ryle.

Possession of skill requires having knowledge states, possession of which yields modal knowledge that yields voluntary control over one's actions. What is the nature of this modality? One might worry that this modality *presupposes* skill. That is, one might worry that one knows that one could shoot an arrow by fitting it into the bow only if one has the *skill* to fit an arrow into a bow, one might therefore worry that an infinite regress of skills threatens. There is no threat of regress. Any interesting skill involves knowledge of what to do initiate activities, where possession of this knowledge requires more basic skills. For example, someone with skill at tennis has skill at serving. But at some point, all such knowledge will rest on knowledge of *basic* actions, such as grasping an object or lifting one's arm. These activities are *not* skills; they are not acquired by or improved upon by training in adult life. Their manifestation is nevertheless under our voluntary control.

Of course, we are not saying that uncontroversial cases of skill *only* require knowing what to do to initiate actions. Typically, the process of becoming more skilled involves learning about multiple actions involved in success at the activity, in addition to their initiation conditions. The same kind of knowledge that is used to initiate an activity can also be injected at anytime in the ongoing course of that activity. For example, a tennis player changes her mind and switches from a groundstroke to a drop shot based on the position of the opponent. Such cases of learning are also knowledge. Our claim now is that it is obvious to the point of not requiring argument that *having a skill requires some knowledge about the activity*. The example of the knowledge we use is the knowledge of what to do to initiate actions of that sort. Surprisingly, as will emerge below, this very uncontroversial claim is sufficient to refute basic presuppositions of neuroscience, such as the view that skill is identical to procedural knowledge.

As far as perceptual ability is concerned, it is considerably less clear that someone with well-trained abilities knows what to do to initiate the action that manifests the ability. Perhaps one may appeal to the element of agency involved in directing oneself to a stimulus, which one may count as initiating an “intentional action” of perceptual acuity. If one does think of perceptual discrimination, e.g., the judgment that three lines are all aligned, as an intentional action on this basis, then an able discriminator of line orientation knows what to do to initiate actions of orientation discrimination. However, the implausibility of this description is clear. It is implausible to think of perceptual discrimination as a kind of action at all, much less an intentional action. The fact that capacities such as perceptual acuity do not characteristically manifest themselves as intentional actions explains why it is incorrect to think of such capacities as skills. We have used *knowing what to do to initiate the action* as a feature of skill that explains the fact that the manifestations of skill are intentional actions. It should therefore be no surprise to discover that abilities possession of which does not require voluntary control seem to be abilities the exercise of which does not seem to require infinitival knowledge, such as knowing what to do to initiate action, or knowing how to do it.

The various improvements that occur with perceptual discrimination of line orientation are not the acquisition and improvement of a skill. It is only in an irrelevant sense that it is true that the able line orientation discriminator knows what to do to initiate line orientation discrimination, and it is quite odd to say that the able line orientation discriminator knows how to discriminate lines. The operations of these faculties somehow reside between Anscombe's example of the peristaltic movement of the gut (Anscombe, 1963) and the operations of genuine skills.

What about learned activities that colloquially clearly seem to fall under the category of skill, yet seem perception-like rather than motor in their deliverances? A clear case is wine tasting. We have argued that skills are manifestations of intentional action but *prima-facie* it may seem that a professional wine taster is merely better at perceiving subtle variations in taste, rather than engaged in activities that could be described as intentional. One might therefore think that our view is incompatible with classifying wine tasting as a skill (though certainly it is not a motor skill). However, the *prima facie* description of wine tasting that leads to this objection is flawed. Professional wine tasting (and even more obviously radiology) involves active decision-making. It requires deciding between relevant factors in making an overall judgment. A professional wine taster can certainly make intentional errors in the course of delivering her judgment. Since this sort of active decision-making is paradigmatically intentional, classifying wine tasting as a skill is consistent with the view we are taking here on the general nature of skill.

In philosophical discussions of skill dating back to the ancients, it has been characterized as a capacity that one possesses only if one can make voluntary errors. This is a feature that is well explained by the view that skills directly manifest in intentional actions. We have argued that the paradigm, uncontroversial cases of skill are like this, and in all such cases, it is also clear that knowledge about an activity is required in order to possess even a minimal level of skill. Kinds of perceptual acuity lack important features that we ascribe to skills; in particular knowing what to do to initiate the action. We have also argued that the feature of skill to which Aristotle draws our attention is explained by the hypothesis that knowing what to do to initiate the action is required for even a minimal level of skill.

However, we now face a worry. Not all agents who possess such skills are able to *verbally articulate* their knowledge of what to do to initiate an action. Is this an objection to our thesis that any uncontroversial case of skill requires knowing facts about that activity? What is the connection between knowing something and being able to verbally articulate its content?

## Propositional vs. declarative knowledge

It is well known that many experts at any activity are not the best at explaining facts about the activity. If Socrates were correct in the *Gorgias* that expertise requires the ability to explain to others the first principles of a craft, then many people we regard as experts would merely have a habit and not a skill (Plato, 1998). Does our second necessary condition on having skill at φ-ing, where φ-ing is a skill in the sense of paradigmatic examples like playing the piano and playing chess, that it requires knowledge of what to do to initiate action of φ-ing, commit us to the consequence that someone who has skill at φ-ing can explain how to φ, or at the very least explain what to do to initiate an action of φ-ing?

Fodor (1968, p. 70) formulates this precise objection in his discussion of the “intellectualist” view attacked by Ryle (1949), that knowing how to do something is a kind of knowing that:

There is, for example, the distinction between what we know how to do and what we know how to explain. The cases that come to mind here are skills, and the relevant gossip is that the best practitioner need not be the best teacher.

The intellectualist holds that at least part of skill is propositional knowledge. In fact, some intellectualists, such as Carlotta Pavese (forthcoming) argue that *all* skill is knowledge. Do views such as ours, according to which a large part of skill is knowledge, and views such as Pavese's, according to which all of skill is knowledge, entail that the knowledge in question is verbalizable?

As Fodor explains, possession of knowledge does not entail that the skilled agent needs to be able to *explain* that knowledge:

There is a real and important distinction between knowing how to do a thing and knowing how to explain how to do that thing. But that distinction is one that the intellectualist is perfectly able to honor. Dogs, cats, and preverbal children know how to do all sorts of things they can't explain how to do; the ability to give explanations is itself a skill – a special kind of knowing how, which presupposes general verbal facility at the very least. But what has this to do with the relation between knowing how and knowing that? And what is there here to distress an intellectualist? (Fodor, 1968, p. 71) (Stanley (2011a) shows how a propositional view of knowing how invalidates the inference from “x knows how to F” to “x knows how to explain how to F.”)

Fodor's point is that having propositional knowledge is not connected with the ability to articulate one's propositional knowledge.

Cognitive neuroscientists postulate a category they call “declarative knowledge,” which is often represented as coinciding with the notion of propositional knowledge in (say) epistemology. It is a requirement on declarative knowledge that it is verbally articulable. However, it is not clear what is meant by “articulable.” Stanley (2011a, pp. 157–163) argues that the claim that all propositional knowledge is articulable is straightforwardly false, or that the requirement of being capable of verbal articulation can be so easily met to do the work cognitive neuroscientists think it does. It is worthwhile rehearsing the argument.

Stanley (2011a, p. 161) considers an expert yet punch drunk boxer, who is skilled at fighting a southpaw, but cannot give an informative description of how he does it. Nevertheless, the boxer can articulate his knowledge; by swinging his fists while facing a southpaw, he can say “this is how you fight a southpaw.” If verbal articulation excludes descriptions containing words like “this,” then it is easy to show that not all propositional knowledge is capable of verbal articulation. Looking at an edge of a mysterious object, most of which is hidden from view, I can come to know that *this object is partially hidden from view* (thanks to Carlotta Pavese for this example). But since I don't know the nature of the object, this is propositional knowledge that cannot be verbally articulated without using words like “this” and “that.” So if “capable of verbal articulation” excludes verbal articulation with demonstratives like “this” or “that,” then it is clear that not all propositional knowledge is capable of verbal articulation. But if sentences containing words like “this” and “that” are allowed to be examples of successful verbal articulation, then stock examples of cases in which one's procedural knowledge is not supposed to be capable of verbal articulation fail to be examples of this sort. It's simply not clear what is meant by the notion of verbal articulation that underlies talk of declarative knowledge.

Even supposing declarative knowledge to be a well-defined notion, it just is not a well-motivated requirement on knowledge of facts. Humans are among the few species with a linguistic capacity. Our linguistic capacity gives us vast resources. It gives not only the ability to communicate complex messages to one another, but also allows us to access concepts that would otherwise be inaccessible without this ability. But there is simply no reason to conclude that concept possession *generally* requires the capacity to linguistically articulate. There is no reason to deny that at least some animals without a capacity to verbally articulate their content nevertheless possess the same concepts we do. The capacity to have attitudes to propositions generally does not depend on the capacity for verbal articulation.

Why have so many philosophers and scientists wrongly thought that knowing a fact requires the ability to verbally articulate it? This is because some examples of knowledge do seem to be ones that characteristically manifest themselves in verbalization. But to take this as a requirement on factual knowledge generally is to mistake a property of some instances of a type with an essential property of the type. As Wittgenstein so elegantly characterizes the error in the *Philosophical Investigations* (1953):

78. Compare ‘knowing’ and ‘saying’:

How many feet high Mont Blanc is –

How the word ‘game’ is used –

How a clarinet sounds

If you are surprised that one can know something and not be able to say it, you are perhaps thinking of a case like the first. Certainly not of one like the third.

There is also no compelling reason that has emerged in the theory of knowledge over the centuries to think that the capacity to entertain the attitude of *knowledge* towards a proposition requires verbal articulation. According to *internalism about knowledge*, it is a requirement for an agent to know that *p* that agent's *reasons for believing that p* are accessible to her. Internalism about knowledge is a controversial doctrine; externalists hold that one can know that *p* without remembering one's reasons for believing that *p*. For example, to take a famous example from Alvin Goldman, many people know that Lincoln was President during the Civil War without remembering where they learned it. Nevertheless, even internalists about knowledge do not explain the accessibility of reasons in terms of the capacity to verbally articulate those reasons. If even the most intellectualist view in epistemology does not require what is accessible to the agent to be verbally articulable, it is difficult to see how one could begin to motivate the view that knowledge of facts requires an agent to have the capacity to verbally articulate those facts.

Robert Stalnaker (2012, p. 755) writes:

In some cases, knowledge may be manifested in the assertion and defense of what one knows, but in general, propositional knowledge is the possession of information and the capacity of to use that information to guide one's actions. The action of asserting what one knows is just one special case of an action that manifests one's cognitive capacity.

We have argued that there is no persuasive reason from the philosophical literature on knowledge to take verbal articulation of its content to be a condition on knowledge. To make this point, one does not need to adopt a positive account of the nature of knowledge. Nevertheless, we find much wisdom in Stalnaker's remark.

On the hypothesis that one should not give necessary and sufficient conditions for anything, we have not attempted to do so either for skill or knowledge. Instead, we have made two claims about having skill at φ-ing, only the first of which we are committed to holding universally (i.e., across all skills). The first is that having skill at φ-ing requires being trained past baseline. The second is that there is a large class of skills, the ones the manifestations of which are intentional actions, such that having skill at φ-ing requires knowing what to do to initiate an act of φ-ing, or more colloquially, how to start φ-ing (knowledge that may be different in different circumstances). In this section we have argued that the fact that some agents (and perhaps animals too) cannot verbally articulate this knowledge is no obstacle to its status as knowledge. Declarative knowledge may not even be a well-defined notion, and even if it were, in its broadest sense, it does not capture knowledge of facts.

We now turn to the case of HM, and more generally, the notion of procedural knowledge.

## The lessons of HM: motor acuity, action selection and knowledge

The case of HM is seminal for field of cognitive neuroscience, specifically the idea that there are multiple memory systems in the brain (Cohen and Squire, 1980). HM was a patient with intractable epilepsy who underwent bilateral temporal lobectomy and was subsequently found to have persistent and pervasive anterograde amnesia—he would rapidly forget events soon after they occurred. In a groundbreaking experiment, the psychologist Brenda Milner had HM perform a mirror drawing task in which he had to trace the outline of star with a pencil through a mirror with vision of his own arm obscured (Milner, 1962). HM showed improvement over 3 days on this task even though on each day he had no explicit memory for ever having encountered the task before nor even a feeling of familiarity with it. This is an admittedly fascinating and important result. However, it has also done great harm. It is partly responsible for the overly simplistic mapping between the declarative/procedural distinction and other distinctions such as that between knowledge and skill, or between theoretical and practical capacities. As we argue here the case of HM, and patients like HM, simply does not support drawing such distinctions.

Here is a fact about HM. Each time HM performed the task he received *explicit verbal instruction*, and was able to use that knowledge each time. HM of course forgot that he had used explicit knowledge. But that of course does not entail he did not require the knowledge at the time. To understand what the original results do or do not mean, it is useful to consider more recent experiments conducted in patients with similar medial temporal lobe lesions to HM since the 1960s. The general approach in follow-up studies in patients with medial temporal lobe lesions, as in the original Milner experiment, is to demonstrate dissociation between improvement in motor performance variables, usually time to completion and error/accuracy measures, and ability to explicitly recall aspects of the task. What becomes apparent when considering this literature is that the amnestic patients could not perform any of the tasks *unless instruction was provided on each day*.

For example, Shumita Roy and Norman Park introduced amnesic patients to novel tools the function of which could only be known by being told; they could not be solved through simple affordance or mechanical problem solving (Roy and Park, 2010). Critically although the patients did improve on motor performance variables across days, this only happened in the context of explicit instructions about the tool and how to use it *every day*. If the motor skill is considered the *combination* of knowing what to do along with doing it better then the idea that the motor skill *in toto* is procedural knowledge is doubly misleading. It is misleading because it is obvious that the patients need instruction to even get started at the activity. Surely, someone who has skill at an activity does not require such instruction. A tennis match would not proceed very well if the player needed a continuous stream of instructions as to what a tennis racket is, what a backhand is, what a ball toss is, what the point of the game is etc.

It is also misleading because the component of novel tool use that is being learned across days in these patients is not knowledge at all. Instead, it is a kind of learning directly analogous to perceptual learning. Shmuelof and colleagues have recently coined the term “motor acuity” to describe practice-related reductions in movement variability and increases in movement smoothness (Shmuelof et al., 2012). They have also shown that healthy subjects will adapt to perturbations like a mirror despite it being contrary to their own intentions (Mazzoni and Krakauer, 2006). Such adaptations are not the acquisition of something that characteristically manifests in intentional action, i.e., they are not the acquisition of skills. The point we have here been making is that performing a skilled motor action in *any ordinary sense* (where the paradigm cases are activities like tennis, cooking, dancing) centrally involves propositional knowledge. Another critical point is that if healthy subjects were to continue practicing with the Park and Roy tools, some might adopt better strategies than others—for example explicitly noticing that if they adopt a particular posture and arm configuration that they use the tool better. This is explicit knowledge that could either be coached or acquired just through looking and playing with the tools over time. This knowledge would combine with acuity for more skilled performance overall. In this way healthy subjects would outperform the patients over time because they can accumulate knowledge about the task and select more optimal actions even if they are matched in terms of ability to improve acuity of any given action.

It is clear from the literature in cognitive neuroscience that in the rush to ground an apparently significant theoretical distinction in the brain, such as the distinction between knowledge and skill, or the theoretical and the practical, the point that motor skill requires knowledge has been largely missed (but see Bruner, 1973; Willingham, 1998). Motor skills have been incorrectly identified with the part of skill that is not knowledge. As Roy and Park (2010) write, in the very same paper that raises serious problems for the identification of motor skill with procedural knowledge “It has been proposed that some aspects of tool-related knowledge (e.g., tool attributes) may rely on the declarative memory system, while other aspects of tool knowledge (e.g., motor skills) appear to be represented in the procedural memory system.” Roy and Park then argue that so-called “tool knowledge” is not motor skill, because it involves both declarative and procedural aspects. But this is a bizarre conclusion to draw and coming to it again indicates the tendency to want to exclude a declarative component from motor skill. Obviously, tool knowledge is a paradigm example of motor skill; tool use is for example Heidegger's central example of a motor skill. The correct conclusion to draw from Roy and Park (2010) is rather that *motor skill* involves both a knowledge component and a component that is not knowledge-based, namely the so-called procedural aspect.

One might think that one can still use these facts from cognitive neuroscience to ground colloquial or folk distinctions between knowledge and skills, or the theoretical and the practical. For example, one might maintain that manifesting skills involves both a knowledge component and a “procedural” component, whereas manifesting knowledge does not. However, this is not correct. One can make this point very simply. All sorts of cognitive processes that do not involve knowledge are implicated in the manifestation of knowledge. For example, the acquisition of perceptual knowledge requires perceptual ability. Perceptual ability is not a kind of knowledge. It follows that manifesting perceptual knowledge, say by telling someone the color of a desk, requires perceptual acuity. Nevertheless, telling someone the color of a desk is clearly the manifestation of knowledge of facts (Stanley, 2011a, pp. 171–172).

The point also holds for the manifestation of quintessential cognitive skills like chess, which also improves with practice. A chess player makes explicit decisions based on explicit knowledge of the rules and of previous games, but there is considerable evidence that higher-level cognitive processes can be influenced by unconscious information processing (van Gaal et al., 2012). Similarly, the mathematician makes explicit decisions based on explicit knowledge of rules. But the manifestation of this knowledge involves much implicit processing of which she is unaware.

The moral of the foregoing is that there is in fact no difference between practical and theoretical tasks with respect to the *kind* of knowledge. Motor skill tasks have an acuity component that is directly analogous to perceptual acuity. But neural computations equivalent to those underlying both perceptual and motor acuity are no doubt implicated in many theoretical pursuits as well. One might perhaps attempt to ground an interesting distinction between theory and practice, or between theoretical knowledge and skill, by appeal to different kinds of content in the relevant knowledge states. However, this is a hopeless task. The knowledge involved in having skill at tennis is different than the knowledge involved in being a math professor. But for all we know the content of the knowledge involved in being a French professor is more similar to the content of the knowledge involved in having skill in tennis than it is to the knowledge involved in being a math professor.

Manifesting skill is more than just adaptation or improved motor acuity. It involves a large amount of propositional knowledge about the relevant activity. It is also quite plausible that in the typical case manifesting propositional knowledge also involves perceptual and motor components. There is a distinction between perceptual and motor ability, on the one hand, and knowledge, on the other hand (though possession of the latter in many cases requires the possession of the former). But it is simply hopeless to ground any interesting philosophical distinction between skill and something else on the distinction between perceptual and motor acuity, on the one hand, and propositional knowledge, on the other. Manifesting any kind of knowledge, and any kind of skill, requires possession of both.

We have not delineated in this article exactly how propositional knowledge leads to selection of the optimal actions for a particular task. Coaching involves verbal instruction and demonstration to convey a mean single action (e.g., a backhand) or sequence of actions (e.g., weaving) to perform the task. Our core claim is that real world motor skills require the employment of the correct average action, presumably selected from a large potential repertoire through external instruction or self-instruction based on ever-accumulating knowledge of the task, followed by increased precision of the selected action through practice (motor acuity). Thus sport and other activities that are generally considered skilled require knowledge as a scaffold for subsequent development of acuity of the selected action components. We should also emphasize that use of knowledge to select actions continues in the skilled state because there is always the possibility to perform new actions based on further knowledge and then develop acuity at these new actions (the Fosbury flop in high jump would be an example of this). In addition, the ability to select the right action from a repertoire based on context also improves with experience and knowledge. This is congruent with the observation that athletes are not paying less attention than novices when they play and are more aware of errors.

Does the fact that manifesting skill requires knowledge preclude non-human animals from possessing skills? We are agnostic as to whether animals can be skilled. It is possible that as a task is weighted increasingly toward rules, alternative actions, and on-the-fly problem solving, then simple operant conditioning may not suffice to accomplish the task. In this sense non-human animals may be limited in a way similar to the amnestic patients in the Roy and Park experiment. Although non-human animals may exhibit the same behavior as humans, this does not entail that the explanation for the behavior is the same. It could be that the explanation skilled action in humans involves intellectual capacities lacking in non-human animals. As Ryle (1949, pp. 128–129) writes:

To say that John Doe can swim differs from saying from saying of a puppy that it can swim. For to say that the puppy can swim is compatible with saying that it has never been taught to swim, or had practice in swimming, whereas to say that a person can swim implies that he has learned to swim and has not forgotten. The capacity to acquire capacities by being taught is not indeed a human peculiarity. The puppy can be taught or drilled to beg, much as infants are taught to walk and use spoons. But some kinds of learning, including the way in which most people learn to swim, involve the understanding and application of spoken instructions or at least of staged demonstrations; and a creature that can learn things in these ways is unhesitatingly conceded to have a mind, where the teachability of the dog and infant leaves us hesitant whether or not to say that they yet qualify for this certificate.

Alternatively, it could be that animals can both possess concepts and bear the knowledge relation to propositions (if so, one would need to explain why animals cannot acquire certain skills that humans can; perhaps because there complex skills require complex concepts, which cannot be grasped by animals). Obviously, deciding between these options is beyond the scope of this paper.

So what, then, are motor skills? We have not taken a final position here. Our point has been rather to argue that manifesting one's skill at an activity requires a large amount of ever-accumulating propositional knowledge about that activity. This point is consistent with those views of the nature of skill that assigns a central role to knowledge of facts. For example, Carlotta Pavese argues that skills are knowledge states (Pavese, Unpublished). Peter Railton argues that skills are belief states (Railton, forthcoming). Stanley (2011a, pp. 181–184) suggests two distinct accounts of skill, neither of which identifies skills with cognitive states, yet nevertheless accords a central guiding role to such states. According to the first, having a skill is having a state that yields the “fluid acquisition of reasons for acting in novel situations,” where reasons for acting are knowledge states (see Stanley and Williamson (forthcoming) for development of this view). According to the second, as delineated by us here, skills are composite states, requiring both increasing knowledge of required actions, and practice-related improvement in the selection and acuity of these actions. All of these views of skill accord knowledge a central role, and as a result are each consistent with the central moral of our paper.

## Conclusions

Hubert Dreyfus's work on skill, which is influenced by Merleau-Ponty and Heidegger, has been deeply influential in philosophy and the social sciences. Dreyfus has posited what he calls a “five stage model of skill acquisition,” from novice to expert (Dreyfus and Dreyfus, 1980). According to Dreyfus, as one becomes more proficient, one's actions move from being guided by decisions based on knowledge to being rather more like perceptual states. As he writes (Dreyfus and Dreyfus, 1986, p. 371), “Action becomes easier and less stressful as the learner simply sees what needs to be achieved rather than deciding … ” The merely proficient actor falls short of expertise, precisely because “after seeing the goal and the important features of the situation, must still decide what to do. To decide, he falls back on detached rule following.” (Dreyfus and Dreyfus, 1986) The difference between the merely proficient performer and the expert precisely is that the expert no longer needs to make decisions about what to do based on her knowledge about the activity.

The proficient performer, immersed in the world of his skillful activity, sees what needs to be done, but decides how to do it. The expert not only sees what needs to be achieved: thanks to a vast repertoire of situational discriminations he sees how to achieve his goal.

On Dreyfus's view, expert performance is a *species* of perceptual acuity. It does not involve the elements of agency that result from knowledge. We have argued that this is a deeply flawed model of skill possession. It suggests, for example, that an expert tennis player stands to tennis as HM stands to mirror drawing. As we have seen, patients like HM cannot acquire skills with novel tools, precisely because they cannot retain or accumulate the relevant knowledge to guide them. A view of expertise that makes it analogous to motor adaptation of the sort that occurs in the mirror-drawing task on cannot be correct because it leaves out the knowledge component that is required in order for adaptation or acuity to manifest.

Although we have not addressed the topic in depth here, another position taken by Dreyfus is that true expertise is somehow automatic or habitual, and knowledge is no longer required. While it is indeed true that certain motor activities can become habitual or automatic over time, we would again argue that is exactly what does not happen when a motor skill such as tennis or piano is being enacted. In fact the opposite is the case—the musician or athlete is using knowledge of the musical score or the game to dictate to those automatic non-knowledge based components; it is the combination that leads to the skilled performance. Even if it were the case that the need to use knowledge as scaffolding for building certain non-knowledge components diminishes over time once these components are built, this does not imply that new knowledge is not needed to continue acquiring greater skill and add new non-knowledge components. In effect, a continuing symbiosis or bootstrapping between knowledge and non-knowledge is what we propose leads to greater skill overall.

The wide interdisciplinary acceptance of the view that expertise and knowledge are disconnected has caused us to lose our grip on one of the central topics in the history of Western Philosophy. The tendrils of the rich philosophical discussion of skill (*techne*) in Plato and Aristotle are now only recognizable in our intuitive reactions. It remains, for example, considerably more natural to describe someone as skilled at *speaking* French than at *understanding* French. The incorrect identification among neuroscientists of motor skill in its entirety with some of its implicit components has significantly contributed to the problem. We hope to have repaired the damage, by showing that, properly understood, the central discoveries in neuroscience about motor skill lend no support whatsoever to the view that skilled motor activity is not the manifestation of knowledge.

### Conflict of interest statement

The authors declare that the research was conducted in the absence of any commercial or financial relationships that could be construed as a potential conflict of interest.
